# Social interaction reward in rats has anti‐stress effects

**DOI:** 10.1111/adb.12878

**Published:** 2020-01-26

**Authors:** Cristina Lemos, Ahmad Salti, Inês M. Amaral, Veronica Fontebasso, Nicolas Singewald, Georg Dechant, Alex Hofer, Rana El Rawas

**Affiliations:** ^1^ Department of Psychiatry, Psychotherapy and Psychosomatics, Division of Psychiatry I Medical University Innsbruck Innsbruck Austria; ^2^ Institute of Molecular Biology University of Innsbruck Innsbruck Austria; ^3^ Department of Pharmacology and Toxicology University of Innsbruck Innsbruck Austria; ^4^ Institute for Neuroscience Medical University Innsbruck Innsbruck Austria

**Keywords:** cocaine, corticotropin‐releasing factor, p38 MAPK, reward, social interaction, stress

## Abstract

Social interaction in an alternative context can be beneficial against drugs of abuse. Stress is known to be a risk factor that can exacerbate the effects of addictive drugs. In this study, we investigated whether the positive effects of social interaction are mediated through a decrease in stress levels. For that purpose, rats were trained to express cocaine or social interaction conditioned place preference (CPP). Behavioural, hormonal, and molecular stress markers were evaluated. We found that social CPP decreased the percentage of incorrect transitions of grooming and corticosterone to the level of naïve untreated rats. In addition, corticotropin‐releasing factor (CRF) was increased in the bed nucleus of stria terminalis after cocaine CPP. In order to study the modulation of social CPP by the CRF system, rats received intracerebroventricular CRF or alpha‐helical CRF, a nonselective antagonist of CRF receptors. The subsequent effects on CPP to cocaine or social interaction were observed. CRF injections increased cocaine CPP, whereas alpha‐helical CRF injections decreased cocaine CPP. However, alpha‐helical CRF injections potentiated social CPP. When social interaction was made available in an alternative context, CRF‐induced increase of cocaine preference was reversed completely to the level of rats receiving cocaine paired with alpha‐helical CRF. This reversal of cocaine preference was also paralleled by a reversal in CRF‐induced increase of p38 MAPK expression in the nucleus accumbens shell. These findings suggest that social interaction could contribute as a valuable component in treatment of substance use disorders by reducing stress levels.

## Graphical abstract

SI has anti‐stress effects? Rats that express SI CPP, show decreased markers of stress to the level of naive untreated rats. SI preference is potentiated by alpha‐helical CRF (a non‐selective CRF receptor antagonist) but is not affected by CRF icv injections. Cocaine preference is potentiated by icv CRF injections. However, alpha‐helical CRF icv injections or the availability of positive social interaction as an alternative to cocaine, abolish cocaine preference and decrease markers of stress. Thus, SI reward in an alternative context in rats is beneficial against cocaine by reducing stress levels. SI = social interaction; CPP = conditioned place preference; icv = intracerebroventricular; CRF = corticotropin‐releasing factor.

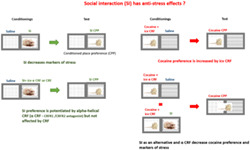

## 1 INTRODUCTION

Switching the preference of a substance‐dependent individual towards non–drug‐related activities remains one of the great challenges that confront drug dependence therapy.^1^ Beneficial social interaction can positively affect personal relationships and may serve as an alternate natural reward to drug use.^1^ We^1^, ^2^ and others^3^ have shown that social interaction with a nonaggressive male in an alternative context blocked cocaine‐induced reinstatement of cocaine conditioned place preference (CPP). These findings suggest that social interaction during extinction, if offered in a context that is clearly distinct from the previously drug‐associated one, may profoundly decrease the incentive salience of drug‐associated contextual stimuli.

It has been repeatedly reported that stress is considered a risk factor that can render individuals more vulnerable to the effects of drug abuse and to relapse after periods of abstinence.^4^, ^5^ It is also well shown that corticotropin‐releasing factor (CRF), one of the major effectors of stress,^6^ promotes drug addiction.^6^ Indeed, CRF increases locomotor responsivity,^7^, ^8^, ^9^ mediates the response to drug‐associated stimuli,^10^ and facilitates stress‐induced relapse to drug seeking.^11^, ^12^, ^13^ This peptide and its receptors CRF1R and CRF2R are expressed in the hypothalamus, as well as in limbic brain areas including amygdala and bed nucleus of stria terminalis (BNST).^14^, ^15^

Several studies also showed that p38 MAPK (MAPK14) is involved in stress and anxiety. Indeed, p38 MAPK was reported to be activated after stress^16^, ^17^, ^18^ in many different regions of the brain including the prefrontal cortex, nucleus accumbens, and the hippocampus. Other studies reported that p38 MAPK activation is involved in the effects of drug of abuse. It has been shown that morphine CPP induced p38 MAPK activation in the nucleus accumbens microglia.^19^ In addition, injections of the p38 kinase inhibitor SB203580 in the nucleus accumbens impaired amphetamine CPP^20^ and morphine CPP.^19^ Previously, we investigated whether cocaine CPP increases p38 MAPK activation in the nucleus accumbens as compared with social interaction CPP. Indeed, control saline rats receiving saline in both compartments of the CPP apparatus and cocaine CPP rats showed similarly enhanced p38 MAPK activation in the nucleus accumbens shell as compared with naïve and social interaction CPP rats.^21^ These results suggest that cocaine per se does not increase p38 MAPK activation in the nucleus accumbens shell and that social interaction reward decreases p38 MAPK activation to the level of naïve rats.^21^

An important question that needs to be addressed is whether the beneficial effects of social interaction reward are mediated through a reverse of stress levels. For that, we analysed stress markers specifically altered behavioural transitions of grooming, urine corticosterone levels, and CRF expression, after cocaine or social CPP expression in rats. With the purpose of examining whether social interaction CPP could also be modulated by the CRF system, we investigated the modulation of CPP to cocaine and social interaction by intracerebroventricular (icv) injections of CRF or alpha‐helical CRF, a nonselective antagonist of CRF receptors. In order to check for potential beneficial protective effects of social interaction against drug abuse and stress, we investigated the availability of social interaction as mutual choice in an alternative context on CRF‐induced effects on cocaine preference and on the subsequent p38 MAPK activation in the nucleus accumbens shell.^21^ Finally, we assessed the effects of icv injections of p38 MAPK inhibitor, SB203580, on the acquisition of concurrent CPP in which rats receive cocaine conditionings in one compartment and social interaction conditionings in the other compartment of CPP to explore whether p38 MAPK inhibitor could shift the preference towards social interaction and promote social interaction reward.

## 2 MATERIALS AND METHODS

### 2.1 Animals

Male Sprague Dawley rats (6 to 8 weeks old; Janvier Labs, France) were housed at controlled environmental conditions (12‐hour light/dark schedule) with food and water supplied ad libitum. Experiments were performed during the light phase of the circadian cycle. All animals were group‐housed until 10 days before the start of the behavioural experiments, from which time on they were single housed.

The Austrian National Animal Experiment Ethics Committee approved all experiments in the present study (BMWF‐66.011/0112‐WF/V/3b/2015).

### 2.2 Conditioned place preference

The CPP apparatus (64 cm wide × 32 cm deep × 31 cm high) is made of unplasticized polyvinyl chloride and consists of a three‐compartment chamber with a middle (neutral) compartment (10 × 30 × 30 cm) connected to the two outer/conditioning compartments (25 × 30 × 30 cm each). The compartments differ in floor (white floor in the middle compartment/stainless steel floor with either holes or stripes in the two conditioning compartments) and in wall (white wall in the middle compartments/vertical or horizontal black and white stripes in the two conditioning compartments).

The acquisition protocol^1^ started with a preconditioning preference test on day 1 (pretest). During the pretest, the animals were allowed to freely explore the three compartments of the apparatus for 15 minutes (no difference was found in the time spent between in the horizontal vs vertical compartment of the CPP during the pretest ‐ Figure S2). The conditioning procedure was performed on eight consecutive training days (days 2 to 9) following an alternate‐day‐design with one training session per day (15 minutes) for a total of four training sessions for each condition: cocaine (hydrochloride salt, corresponding to 15 mg/kg pure cocaine base in a volume of 1 mL/kg saline) or social interaction. Social interaction conditioning consisted of a 15‐minute period in one of the CPP compartments with a weight‐ and gender‐matched conspecific immediately after an intraperitoneal (i.p.) injection of 1 mL/kg saline to control for possible handling and i.p. injection effects. The conspecific remained the same social partner for a total of four periods. Cocaine or social stimulus was paired with the less preferred chamber during pretest. The other chamber was paired with saline. The saline control group received saline in both compartments of the CPP. The CPP test was carried out on day 10, ie, 24 hours after the last conditioning session, in which the rats were placed in the middle neutral compartment and allowed to explore the apparatus for 15 minutes.

In the concurrent CPP paradigm, the protocol started with a pretest session on day 1 followed by eight consecutive training days in an alternate‐day‐design, one 15‐minute training session per day, a total of four training sessions each for social interaction in one compartment or cocaine in th7e other compartment of the CPP, then a CPP test on day 10.

Time that animals spent in each compartment in the pretest and test was recorded and evaluated using Any‐Maze Software.

Preference for a stimulus was defined by the time spent in the stimulus‐associated compartment during the test minus the time spent in the stimulus‐associated compartment during the pretest (preference score). For the saline control group, the preference score was calculated as the time spent in the less preferred compartment during the test minus the time spent in the less preferred compartment during the pretest.

### 2.3 Incorrect transitions of cephalocaudal grooming progression

Grooming is an important element of rodent behaviour with a general pattern of cephalocaudal progression (paw licking→nose/face wash→body wash→tail/genitals wash). Incorrect transitions of cephalocaudal progression between different grooming patterns can be used as a stress marker in rats.^22^ The grooming stages are defined as no grooming (0), paw licking (1), nose/face/head wash (2), body grooming (3), leg licking (4), and tail/genitals grooming (5). The percentage of incorrect transitions were analysed between patterns. Correct transitions between grooming stages include the following progressive transitions: 0‐1, 1‐2, 2‐3, 3‐4, 4‐5, and 5‐0. According to Kalueff et al,^23^ four main types of incorrect transitions include aborted, prematurely terminated (eg, 3‐0 and 4‐0), skipped (eg, 1‐5 and 2‐5), reversed (eg, 3‐2, 4‐1, and 5‐2) and incorrectly initiated (eg, 0‐4 and 0‐5). Analyses were done during the test session of CPP for each treatment condition. Naïve values were evaluated during the pretest session of the CPP before the animals underwent conditionings.

### 2.4 Urine corticosterone measurements

Twenty‐four hours after the CPP test,^21^ urine from rats was collected and quantitative corticosterone/creatinine measurements were performed using the corticosterone ELISA Kit (Abcam) according to the protocol provided with the kit. For naïve values, urine from naïve group‐housed nontreated rats served as baseline level.

### 2.5 Quantitative real‐time polymerase chain reaction

Twenty‐four hours after the CPP test, rats were sacrificed by an overdose of isoflurane. This time point was chosen according to our previous findings^21^ in which social interaction reward decreased p38 MAPK activation to the level of naïve rats in the nucleus accumbens shell, only 24 hours after the CPP test.

Brains were removed and immediately frozen in −40°C isopentane. The amygdala, BNST, the hypothalamic paraventricular nucleus, and the nucleus accumbens shell were punched out using a sample corer (Fine science tools, 11G‐17G) while viewing sections using magnifying goggles from thaw‐mounted coronal 200‐μm sections at −15°C in a cryostat.^1^

Total RNA was isolated from the dissected brain regions using Trizol (Invitrogen) according to the manufacturer's recommendations. To avoid contamination with genomic DNA, total RNA was treated with DNase (2 U/μL) using the TURBO DNA‐free Kit (Ambion). RNA was reverse transcribed in the presence of random hexamer primers and MultiScribe Reverse Transcriptase (50 U/μL) in a total volume of 20 μL employing the high‐capacity cDNA reverse transcription kit with RNase Inhibitor (Applied Biosystems).

After dilution with 80 μL of water, 3 μL of the diluted cDNA was used as a template for amplification (duplicates) with AB Fast SYBR Green mastermix (Applied Biosystems). Real‐time polymerase chain reaction (RT‐PCR) quantification was performed on a 7500 Fast Real‐Time PCR system (Applied Biosystems) using the following cycle settings: 20 seconds 95°C, 40 cycles of 95°C for 3 seconds, and 60°C for 30 seconds. All PCR primers were designed using PrimerSelect 5.05 software (DNASTAR). Ct and ΔCt values were calculated by using the 7500 software v2.0.1 and GAPDH as a reference gene.

The primers used in this study are detailed in Table S2.

### 2.6 Surgery and icv injections

Rats were anesthetized with isoflurane. Then, each rat was implanted with a 22‐gauge guide cannula above one of the lateral ventricles. The stereotaxic coordinates used were −0.9 mm from bregma, 1.4 mm lateral from the midline, and −3.0 mm from the skull surface. The incisor bar was set at −3.5 mm. The cannulae were secured by dental cement and anchored to stainless steel screws fixed to the skull. Postoperative analgesia was provided to the animals during the recovery period. The animals were allowed to recover for 6 days from surgery.

Human/rat CRF, alpha‐helical CRF (9‐41), and SB203580 were purchased from Tocris.

CRF (1 μg/rat) was dissolved in physiological saline; alpha‐helical CRF (10 μg/rat) was dissolved in distilled water^24^ with the pH adjusted to 6.7; and SB203580 (1 μg/rat) was dissolved in 100 % DMSO and then diluted in saline so the final concentration of DMSO reached 2%. Vehicle (VEH) injections consisted of physiological saline solution. The drugs were injected icv at a volume of 5 μL.

icv injections of CRF and alpha‐helical CRF were administered 1 hour prior to conditioning. icv injections of SB203580 were administered 30 minutes prior to conditioning.

For cocaine CPP and social CPP, injections were given before cocaine or social interaction conditioning. In control rats, injections were given before conditionings in the less preferred compartment. In concurrent CPP, injections were given prior to both cocaine and social interaction conditionings.

**FIGURE 1:**
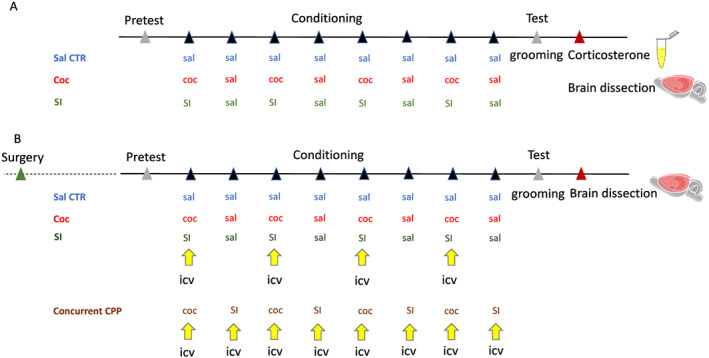
Time line of the experiments performed in the study. A, Animals without surgery. Upon arrival, rats were single housed and got a period of habituation before the start of the experiment. Conditioned place preference (CPP) started with a pretest, followed by eight conditioning days in alternation between coc/sal for cocaine CPP or SI/sal for social interaction CPP and sal/sal for the saline CTR group. During the test, the grooming of each rat was analysed in order to assess the percentage of cephalocaudal progression. Then, 24 hours after, samples of urine were collected from each rat in order to evaluate the corticosterone levels before being sacrificed. Brains were removed, frozen, and then dissected on a cryostat where punches from the bed nucleus of stria terminalis (BNST), amygdala, and hypothalamus were collected for quantitative polymerase chain reaction (qPCR) analysis. B, Animals with surgery. Upon arrival, rats were single housed and got a period of habituation before the start of the experiment. Each rat was implanted with a guide cannula above one of the lateral ventricles and was then allowed to recover. CPP started with a pretest, followed by eight conditioning days in alternation between coc/sal for cocaine CPP or SI/sal for social interaction CPP and sal/sal for the saline CTR group. Before each coc or SI conditioning and saline pairing in the less preferred compartment of CPP, rats received an icv injection of VEH, CRF, or alpha‐helical CRF. In the case of concurrent CPP, in which rats were conditioned with coc in one compartment and with SI in the other compartment, rats received icv injection of VEH or SB203580 before each coc and SI conditioning. During the test, the grooming of each rat was analysed in order to assess the percentage of cephalocaudal progression. Then, 24 hours after, brains were removed, frozen, and then dissected on a cryostat where punches from the nucleus accumbens shell were collected for qPCR analysis. coc, cocaine; icv, intracerebroventricular; Sal, saline; SI, social interaction. The number of animals may sometimes differ across the flow of the experiments because of technical problems such as problem in the dissection of the region of interest for RNA extraction given that the regions of interest are relatively small

A general time line for the experiments performed in the study is represented in Figure 1.

### 2.7 Statistical analyses

All data were expressed as mean ± SEM (standard error of the mean). According to the number of effects implicated, one‐ or two‐way analysis of variance (ANOVA) or two‐tailed unpaired *t* test using Graphpad Prism and Statview programs were used. Results showing significant overall changes were subjected to Tukey's multiple comparison post hoc test. Effect sizes were calculated as Cohen's *d.* The level of statistical significance was predefined at a *P* < .05.

## 3 RESULTS

### 3.1 Social interaction reward decreases stress markers to the level of naïve rats

**FIGURE 2:**
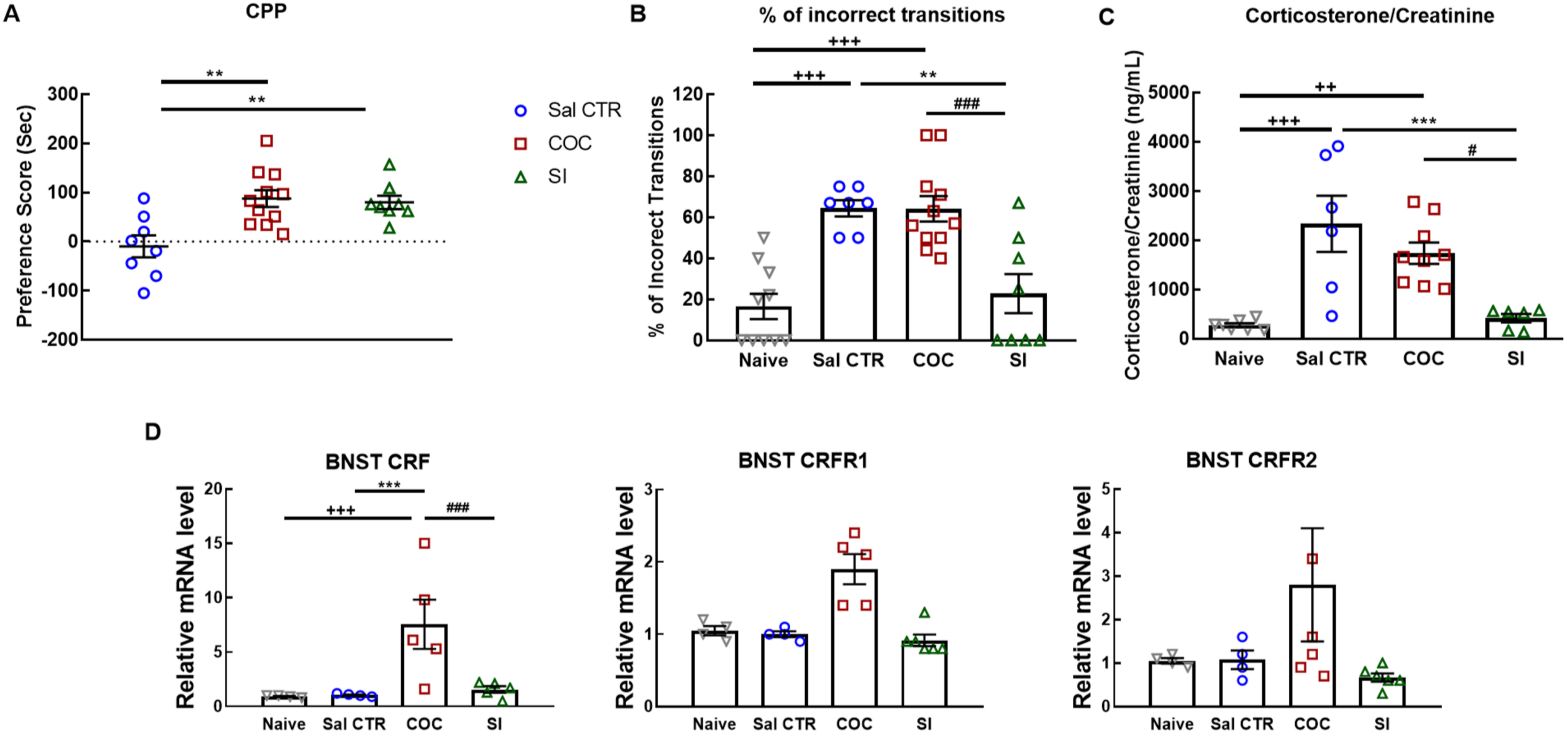
A, Conditioned place preference (CPP) to cocaine or social interaction (n = 8‐11). Saline control rats received saline injections in both compartments of the CPP. Preference score is the time that the rat spent in the stimulus‐associated compartment during the test‐pretest. ^**^ *P* < .01, different from Sal CTR. Sal CTR (n = 8); COC (n = 11); SI (n = 8). B, Percentage of incorrect transitions of cephalocaudal grooming progression (n = 7‐11). Naïve values are calculated during the pretest session; Sal CTR–, COC‐, or SI‐associated percentage of incorrect transitions is calculated during the test session following each treatment, respectively. ^**+++**^ *P* < .001, different from naïve; ^**^ *P* < .01, different from Sal CTR; ^###^ *P* < .001, different from COC. Naïve (n = 10); Sal CTR (n = 7); COC (n = 11); SI (n = 8). C, Corticosterone/creatinine levels in urine of rats 24 hours after the CPP test (n = 6‐9). Naïve rats are untreated group‐housed rats. ^++^ *P* < .01, ^+++^ *P* < .001, different from naïve; ^***^ *P* < .001, different from Sal CTR; ^#^ *P* < .05, different from COC. Naïve (n = 7); Sal CTR (n = 6); COC (n = 9); SI (n = 6). D, Corticotropin‐releasing factor (CRF), CRFR1, and CRFR2 expression in the bed nucleus of stria terminalis (BNST) 24 hours after the CPP test (n = 4‐6). Naïve rats are untreated group‐housed rats. ^+++^ *P* < .001, different from naïve; ^***^ *P* < .001, different from Sal CTR; ^###^ *P* < .001, different from COC. BNST CRF: Naïve (n = 4); Sal CTR (n = 4); COC (n = 5); SI (n = 5); BNST CRFR1: Naïve (n = 4); Sal CTR (n = 4); COC (n = 5); SI (n = 6); BNST CRFR2: Naïve (n = 4); Sal CTR (n = 4); COC (n = 4); SI (n = 4). Statistical test: two‐way analysis of variance (ANOVA) followed by Tukey's post hoc test. Control, Sal CTR; COC, cocaine CPP; SI, social CPP

Rats conditioned to cocaine or social interaction expressed a significant preference to cocaine or social interaction, respectively, as compared with the saline control group receiving saline injections in both compartments of the CPP (one‐way ANOVA, treatment effect, *F*_(2,24)_ = 8.504; *P* = .0016; control [n = 8] vs cocaine CPP [n = 11], *P* = .0021, Cohen's *d* = 4.86; control vs social CPP [n = 8], *P* = .008, Cohen's *d* = 4.84). Cocaine and social interaction had the same reward value as the preference score after cocaine or social interaction conditioning was not different (cocaine CPP vs social CPP, *P* = .9534, ns) (Figure 2A).

After expression of CPP, altered behavioural transitions of grooming, used as a stress marker in rats, were evaluated. The percentage of incorrect transitions in saline control and cocaine CPP groups was increased in comparison to that of naïve rats (one‐way ANOVA, treatment effect, *F*_(3,32)_ = 14.73; *P* < .0001; naïve [n = 10] vs control [n = 7], *P* = .0002, Cohen's *d* = 3.08; naïve vs cocaine CPP [n = 11], *P* < .0001, Cohen's *d* = 2.38). However, the percentage of incorrect transitions in the social interaction CPP group was significantly decreased to the level of naïve rats (control vs social CPP [n = 8], *P* = .0022, Cohen's *d* = 2.04; cocaine CPP vs social CPP, *P* = .0007, Cohen's *d* = 1.72; naïve vs social CPP, *P* = .9163, n.s.) (Figure 2B). The stress hormone, corticosterone, levels in urine were increased in saline control rats and in rats that expressed cocaine CPP as compared with naïve rats and rats that expressed social CPP (one‐way ANOVA, treatment effect, *F*_(3,24)_ = 11.91; *P* < .0001; naïve [n = 7] vs control [n = 6], *P* = .0003, Cohen's *d* = 2.07; naïve vs cocaine CPP [n = 9], *P* = .0037, Cohen's *d* = 2.95). However, corticosterone levels in urine after social CPP were not different from naïve rats (control vs social CPP [n = 6], *P* = .0009, Cohen's *d* = 1.61; cocaine CPP vs social CPP, *P* = .0131, Cohen's *d* = 1.78; naïve vs social CPP, *P* = .9864, n.s.) (Figure 2C). Relative mRNA expression of CRF in the BNST is increased after cocaine CPP as compared with naïve, saline control, and social CPP rats (two‐way ANOVA, treatment effect, *F*_(3,45)_ = 10.12, *P* < .0001; marker effect, *F*_(2,45)_ = 3.735, *P* = .0313; treatment × marker interaction, *F*_(6,45)_ = 2.996, *P* = .0151; naïve [n = 4] vs cocaine CPP [n = 5], *P* < .0001, Cohen's *d* = 1.84; control [n = 4] vs cocaine CPP, *P* < .0001, Cohen's *d* = 1.81; cocaine CPP vs social CPP [n = 5], *P* < .0001, Cohen's *d* = 1.65). Expression of CRFR1 and CRFR2 in the BNST region did not differ among the groups (Figure 2D). CRF, CRFR1, and CRFR2 expression in the amygdala and the hypothalamus was not different among the investigated groups (Table S1).

### 3.2 Social interaction reward is modulated by the CRF system

**FIGURE 3:**
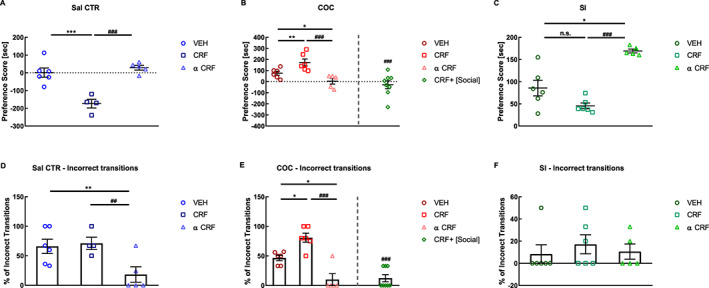
Effects of intracerebroventricular (icv) injections of vehicle, corticotropin‐releasing factor (CRF), and alpha‐helical CRF on saline control, cocaine, and social preference (A‐C) and on associated percentage of incorrect transitions (D‐F) (n = 4‐8). Preference score is the time that the rat spent in the stimulus‐associated compartment during the test‐pretest. CRF+ [social] is a group of rats conditioned with cocaine that received icv injections of CRF prior to cocaine conditioning but also had the opportunity to social interaction in the alternative compartment of the conditioned place preference (CPP). ^*^ *P* < .05, ^**^ *P* < .01, different from VEH; ^##^ *P* < .01, ^###^ *P* < .001, different from CRF. Panel A: VEH (n = 6), CRF (n = 4), alpha‐helical CRF (n = 5); Panel B: VEH (n = 6), CRF (n = 6), alpha‐helical CRF (n = 5), CRF + [Social] (n = 8); Panel C: VEH (n = 6), CRF (n = 6), alpha‐helical CRF (n = 5); Panel D: VEH (n = 6), CRF (n = 4), alpha‐ helical CRF (n = 5); Panel E: VEH (n = 6), CRF (n = 6), alpha‐helical CRF (n = 5), CRF + [Social] (n = 8); Panel F: VEH (n = 6), CRF (n = 6), alpha‐helical CRF (n = 5). Statistical test: two‐way analysis of variance followed by Tukey's post hoc test. α CRF, alpha‐helical CRF; COC, cocaine CPP; Control, Sal CTR; SI, Social CPP; VEH, vehicle

To investigate how the CRF system would modulate cocaine and social CPP, we performed CRF and alpha‐helical CRF icv injections in rats before conditioning to cocaine and social interaction as well as in saline control rats. Injections of CRF and alpha‐helical CRF impacted on the CPP expression of cocaine, social interaction, and saline control (two‐way ANOVA, CPP treatment effect, *F*_(2,40)_ = 42.92, *P* < .0001; icv treatment effect, *F*_(2,40)_ = 4.474, *P* = .0176; CPP treatment × icv treatment interaction, *F*_(4,40)_ = 24.54, *P* < .0001) (Figure 3A‐C).

In saline control rats, icv CRF injections induced a place aversion to the chamber associated with CRF (vehicle [n = 6] vs CRF [n = 4], *P* < .0001, Cohen's *d* = 3.08; CRF vs alpha‐helical CRF [n = 5], *P* < .0001, Cohen's *d* = 5.00). Alpha‐helical CRF injections did not affect the CPP in the saline control group of rats (vehicle vs alpha‐helical CRF, *P* = .6139, n.s.) (Figure 3A). In cocaine CPP rats, icv CRF injections increased cocaine CPP (vehicle [n = 6] vs CRF [n = 6], *P* = .0032, Cohen's *d* = 1.54), whereas icv alpha‐helical CRF injections decreased cocaine CPP significantly (vehicle vs alpha‐helical CRF [n = 5], *P* = .0182, Cohen's *d* = 1.71; CRF vs alpha‐helical CRF, *P* < .0001, Cohen's *d* = 2.73) (Figure 3B). In social CPP rats, icv CRF injections did not affect social CPP (vehicle [n = 6] vs CRF [n = 6], *P* = .3281, n.s.), but icv alpha‐helical CRF injections increased social CPP (vehicle vs alpha‐helical CRF [n = 5], *P* = .0173, Cohen's *d* = 2.64; CRF vs alpha‐helical CRF, *P* = .0004, Cohen's *d* = 9.43) (Figure 3C).

These modulations of CPP were paralleled by changes in the percentage of incorrect transitions of cephalocaudal grooming (two‐way ANOVA, CPP treatment effect, *F*_(2,40)_ = 16.04, *P* < .0001; icv treatment effect, *F*_(2,40)_ = 15.75, *P* < .0001; CPP treatment × icv treatment interaction, *F*_(4,40)_ = 3.970, *P* = .0083) (Figure 3D‐F). In the saline control group, icv CRF injections could not increase further the percentage of cephalocaudal incorrect transitions as compared with vehicle injected rats (vehicle [n = 6] vs CRF [n = 4], *P* = .9315, n.s.). However, icv alpha‐helical CRF injections decreased significantly the percentage of incorrect transitions (vehicle vs alpha‐helical CRF [n = 5], *P* = .0021, Cohen's *d* = 1.63; CRF vs alpha‐helical CRF, *P* = .0021, Cohen's *d* = 2.07) (Figure 3D). In the cocaine CPP group, the percentage of incorrect transitions were increased by icv CRF injections (vehicle [n = 6] vs CRF [n = 6], *P* = .0232, Cohen's *d* = 2.25) and decreased by icv alpha‐helical CRF injections (vehicle vs alpha‐helical CRF [n = 5], *P* = .0212, Cohen's *d* = 2.07; CRF vs alpha‐helical CRF, *P* < .0001, Cohen's *d* = 3.44) (Figure 3E). In the social interaction CPP group, no change was observed in the percentage of incorrect cephalocaudal grooming (vehicle [n = 6] vs CRF [n = 6], *P* = .7593, n.s.; vehicle vs alpha‐helical CRF [n = 5], *P* = .9835, n.s.; CRF vs alpha‐helical CRF, *P* = .8704, n.s.) (Figure 3F).

### 3.3 Social CPP in an alternative context reverses the CRF‐induced increases of cocaine preference

**FIGURE 4:**
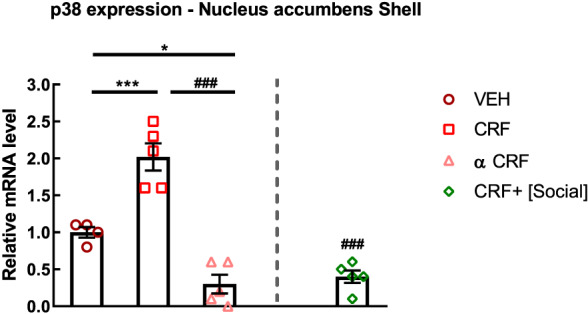
p38 MAPK expression in the nucleus accumbens shell (n = 4‐5). Rats conditioned with cocaine CPP and injected with vehicle, corticotropin‐releasing factor (CRF), or alpha‐helical CRF prior to each cocaine conditioning. CRF + [social] is a group of rats conditioned with cocaine that received intracerebroventricular (icv) injections of CRF prior to cocaine conditioning but also had the opportunity for social interaction in the alternative compartment of the CPP. ^*^ *P* < .05, ^***^ *P* < .001, different from VEH; ^###^ *P* < .001, different from CRF. VEH (n = 4), CRF (n = 5), alpha‐helical CRF (n = 5), CRF + [Social] (n = 5). Statistical test: one‐way analysis of variance (ANOVA) followed by Tukey's post hoc test. α CRF, alpha‐helical CRF; COC, cocaine CPP; Control, Sal CTR; CPP, conditioned place preference; SI, Social CPP; VEH, vehicle

When social interaction was made available in an alternative context, CRF‐induced increases of cocaine preference were reversed completely to the level of rats receiving cocaine paired with icv injections of alpha‐helical CRF (one‐way ANOVA, treatment effect, *F*_(3,21)_ = 8.551, *P* = .0007; CRF vs CRF + [social] [n = 8], *P* = .0005, Cohen's *d* = 3.83; alpha‐helical CRF vs CRF+ [social], *P* = .8829, n.s.) (Figure 3B). This reverse of cocaine preference was paralleled by a reverse in the percentage of incorrect transitions of cephalocaudal grooming (one‐way ANOVA, treatment effect, *F*_(3,21)_ = 22.66, *P* < .0001; CRF vs CRF + [social] [n = 8], *P* < .0001, Cohen's *d* = 3.83; alpha‐helical CRF vs CRF + [social], *P* = .9950, n.s.) (Figure 3E). Furthermore, social interaction as an alternative to cocaine also reversed CRF‐induced increase of p38 MAPK expression in the nucleus accumbens shell region to the level of rats receiving cocaine paired with icv injections of alpha‐helical CRF (one‐way ANOVA, treatment effect, *F*_(3,15)_ = 39.35, *P* < .0001; vehicle [n = 4] vs CRF [n = 5], *P* = .0004, Cohen's *d* = 3.33; vehicle vs alpha‐helical CRF, *P* = .0101, Cohen's *d* = 3.13; CRF vs alpha‐helical CRF [n = 5], *P* < .0001, Cohen's *d* = 4.89; CRF vs CRF + [social] [n = 5], *P* < .0001, Cohen's *d* = 5.09; alpha‐helical CRF vs CRF + [social], *P* = .9418, n.s.) (Figure 4).

### 3.4 P38 inhibitor in a concurrent CPP paradigm does not affect cocaine preference

To investigate whether p38 MAPK inhibitor could shift the preference towards social interaction and promote social interaction reward, icv injections of p38 MAPK inhibitor (SB203580) in a concurrent CPP paradigm, in which rats received cocaine conditionings in one compartment and social conditionings in the other compartment of the CPP, were performed. SB203580 did not induce a shift in preference to any of the compartment associated with either cocaine or social interaction (two‐tailed unpaired *t* test, vehicle [n = 7] vs SB203580 [n = 11], *P* = .6482, *t* = 0.4649, df = 16, n.s.) (Figure S1).

## 4 DISCUSSION

The main finding of the present study is that social interaction reward decreased stress markers at the behavioural, hormonal, and molecular levels. In addition, social interaction CPP was promoted by CRFR antagonist and alpha‐helical CRF but not affected by CRF. Furthermore, social interaction, when available as an alternative choice to cocaine and CRF, was able to reverse not only cocaine preference but also CRF‐induced increased levels of stress.

The percentage of incorrect transitions can be used as a behavioural stress marker in rats behaviour.^22^ The findings of the present study with control rats and cocaine CPP rats performing significantly more incorrect transitions as compared with “naïve” and social CPP rats are in line with our previous findings showing that p38 MAPK activation in the nucleus accumbens shell is increased in control rats and cocaine CPP rats as compared with that in naïve and social CPP rats.^21^ As stress increases the percentage of incorrect transitions in rats,^23^ these results show that social interaction reward decreases a behavioural marker of stress to the level of naïve untreated rats.

Several studies have shown that social stress strongly increases basal corticosterone levels^25^, ^26^ as well as plasma corticosterone response to acute^27^ and chronic stress.^28^, ^29^ We measured plasma corticosterone levels after expression of CPP to social interaction, to cocaine and control rats to check if the anti‐stress effects of social interaction are evident at the stress hormonal level. We found that corticosterone levels are increased in control rats and cocaine CPP rats. This increase was blunted in social CPP rats, supporting the idea that a decrease in stress levels contributes to the positive effects of social interaction.

One of the most important neurotransmitters involved in behavioural components of the stress response is the CRF.^6^ It has previously been reported that social stress induces an increase of CRF mRNA in the dorsal region of BNST and the central amygdala, but not in the paraventricular hypothalamus in rats,^30^ and activates CRFR2‐positive neurons in the medial amygdale.^31^ We now wanted to gain insight into a possible involvement of CRF in effects of social CPP. Indeed, we found that in the BNST, CRF expression was significantly reduced in social CPP as compared with cocaine CPP–expressing rats. In order to elucidate a potential role of the BNST in the protective effect of social interaction on stress, further studies manipulating pharmacologically the BNST should be conducted. However, it appears that CRF expression in the BNST was only increased after cocaine CPP as control rats expressed similar CRF expression in the BNST as naïve rats. Along these lines, an increase in CRF mRNA was found in the BNST of mice with a prior history of cocaine administration.^32^ As CRF expression in the BNST of control rats was significantly lower than in cocaine CPP–expressing rats, the saline control rats may have been subjected to stress that was not sufficient to increase CRF expression in the BNST. However, other stress markers were increased in saline control rats such as p38 MAPK activation in the nucleus accumbens shell,^21^ the incorrect grooming transitions, and corticosterone levels. Moreover, when saline control group received icv CRF injections, rats expressed conditioned place aversion to the context associated to CRF effects. Hence, it seems that endogenous CRF expression requires sufficiently high levels of stress system activation, exacerbated by drugs of abuse,^6^ to be increased in the BNST. However, exogenous CRF was able to induce aversion efficiently in the saline control group.^33^ Cocaine‐induced increase in CRFR1 and CRFR2 did not reach significance in the BNST region.

CRF, CRFR1, and CRFR2 expression in the amygdala and the hypothalamus was not different among the investigated groups. This is in line with other studies showing changes in CRF mRNA only in BNST but not in the amygdala. For example, environmental switch from enriched to standard environment, which produces a negative emotional state, is associated with increased levels in mRNA levels of CRF only in the BNST but not in the amygdala.^34^ Moreover, in a study by McReynolds et al, stress‐induced cocaine reinstatement induced an increase in CRF mRNA in the BNST region but not in the amygdala.^32^ CRF injections in the BNST but not in the amygdala were sufficient to reinstate cocaine seeking.^35^ The same group also reported that injections of CRFR antagonist into the BNST but not into the amygdala completely blocked footshock‐induced reinstatement of cocaine seeking.^35^

In order to investigate the effects of enhancing stress levels on CPP, we investigated the effect of CRF icv injections on saline control, cocaine CPP, and social CPP rats. We found that saline control rats injected with vehicle did not express any preference for any of the compartments of the CPP. However, saline control rats that received icv CRF expressed conditioned place aversion to the compartment associated with CRF injections as shown by Cador et al.^33^ Alpha‐helical CRF injections in saline control rats did not produce place preference or aversion as reported by Lu et al.^36^ Saline control rats injected with vehicle performed 66% ± 12% of incorrect transitions of cephalocaudal grooming, comparable with that of the saline control group in Figure 2B. However, CRF icv injections in saline control rats could not further significantly increase the percentage of incorrect transitions. It seems that stress levels in the CRF‐injected group reached a ceiling effect that can be completely reversed if rats received icv alpha‐helical CRF.

The CRF system has been shown to have major roles in drug‐seeking behaviour.^37^ In addition, it has been shown to be critically involved for the stress‐induced increases in cocaine CPP.^34^, ^38^ Interestingly, long‐term, postnatal CRF overexpression increases cocaine CPP in mice with low behavioural reactivity to novelty associated with heightened anxiety‐like behaviour.^39^ Consistent with these findings, our results show that cocaine CPP is increased after icv injections of CRF before cocaine conditioning. As opposed to CRF effects on behaviour, we found that CRF receptor antagonist prior to each cocaine conditioning blocked cocaine CPP as previously reported by Lu et al,^36^ possibly through a reduction in the extracellular dopamine levels of nucleus accumbens and ventral tegmental area in response to the injection of cocaine.^36^ In addition, icv injections of alpha‐helical CRF significantly attenuated the reactivation of CPP induced by cocaine priming or footshock stress.^10^ The percentage of incorrect transitions of grooming in the cocaine CPP rats injected with vehicle was not significantly different from that of cocaine CPP–expressing rats. Changes observed on the effects of icv injections on cocaine preference were paralleled by alterations on stress levels as assessed by altered behavioural sequencing of grooming. Indeed, levels of stress were increased in rats receiving icv CRF but completely abolished in rats receiving alpha‐helical CRF injections prior to cocaine conditioning.

On the other hand, pharmacological activation of the CRF system has been shown to decrease social interaction.^40^, ^41^ Unexpectedly, the effects of icv CRF injections on the reduction of social interaction CPP did not reach significance as if social CPP was resistant to the effects of CRF. One possibility is that the antistress effects of social interaction reward have antagonized the impact of CRF injections. Indeed, stress levels did not increase after CRF icv injections prior to each social interaction conditioning as assessed by the percentage of incorrect transitions. At the opposite, data from the literature have shown that CRF receptor antagonists increase social interaction.^42^, ^43^, ^44^ Similarly, we found that alpha‐helical CRF icv injections potentiated social CPP. Social CPP–expressing rats displayed a very low percentage of incorrect transitions reflecting low levels of stress that remained unchanged with icv injections.

When drug and social interaction rewards were presented as a series of mutually exclusive choices, it was reported that operant social reward eliminated drug self‐administration, even in rats that met criteria for addiction.^45^ Also, using CPP paradigm, concurrent conditioning for social interaction in one compartment and cocaine in the other compartment, both stimuli produce equal CPP.^1^ These findings suggest that social interaction and cocaine reward have similar conditioned reward value. Recently, it was found that the presence of social interaction during cocaine‐induced CPP test was able to reduce cocaine preference in mice.^46^ These findings propose that when a social interactive stimulus is present as a choice to drugs, it is able to decrease the salience of drug‐associated contextual stimuli.^46^ We found that when social interaction was made available in an alternative context to cocaine, CRF‐induced increases of cocaine preference were reversed completely to the level of rats receiving cocaine paired with icv injections of alpha‐helical CRF. This reversal of cocaine preference was also paralleled by a reversal in altered behavioural sequencing of grooming and in CRF‐induced increase of p38 MAPK expression in the nucleus accumbens shell. These results suggest that the availability of social interaction as an alternative choice was able to abolish drug preference even when it was enhanced by CRF, by lowering stress markers and generating a more resilient phenotype against drugs of abuse and stress. Not much data have been generated about the effect of CRF system on p38 MAPK in the brain. Our results show that the modulation of CRF system has a direct impact on p38 MAPK expression in the nucleus accumbens shell. Indeed, p38 MAPK expression was increased after icv CRF injections prior to each cocaine conditioning and was decreased after icv injections of alpha‐helical CRF prior to each social conditioning.

Previous studies have shown that p38 MAPK antagonists impair the amphetamine‐induced CPP^20^ and that microinjection of p38 MAPK inhibitor SB203580 into the nucleus accumbens prevented the acquisition of morphine CPP.^19^ Given the implication of p38 MAPK activation by stress, we expected that the p38 inhibitor would shift the preference of rats towards the social interaction–associated compartment in the concurrent CPP paradigm. However, we found that icv injections of SB203580 had no overall effects on concurrent CPP with half of the animals preferring the cocaine associated compartment and the other half preferring the social associated compartment. There are a number of differences between the above‐mentioned studies and ours, from the differences in drugs used, in the paradigm used (concurrent CPP vs normal CPP), and the injection site of SB203580 (icv vs locally into the nucleus accumbens). Nevertheless, one study reported that mice receiving SB203580 intraperitoneally before the postconditioning test day exhibited significantly reduced CPP to cocaine.^47^ Hence, it appears that general p38 MAPK inhibition may affect the expression rather than the acquisition of cocaine CPP.

Stress is a key risk factor in the development of addiction and in addiction relapse, and hence treatment failure.^48^ While it may not be possible to eliminate it, stress management through social network strengthening seems to be a good option to prevent and overcome addiction. Generally, positive social support can have a buffering effect on stress responses,^49^ resulting in reduced effects of drugs of abuse. Indeed, social support in all its facets (informational, material, and emotional) is considered as a key external factor for resilience against substance use.^50^ These facets of social support can be facilitated and maintained by different systems, including family, positive peer connections, and community engagements.^49^ The findings of this study give evidence that social interaction could contribute as a valuable component in treatment of substance use disorders by reducing stress levels.

## AUTHORS' CONTRIBUTION

CL and AS performed the experiments with the help of IMA and VF. RER and CL designed the experiments and interpreted the data. RER analysed the data and drafted the manuscript. NS, GD, and AH provided critical revision of the manuscript for important intellectual content. All authors critically reviewed content and approved final version for publication.

## DISCLOSURE/CONFLICT OF INTEREST

The authors declare that they have no conflicts of interest.

## Supporting information

Figure S1: Effects of icv injections of SB203580 on concurrent CPP (n=7‐11). Rats received icv injection of either VEH or SB before each cocaine and social interaction conditioning. Preference score is the time that the rat spent in the cocaine‐associated compartment during the test – pretest. VEH (n=7); SB (n=11). Statistical test two‐tailed unpaired t‐test. VEH= Vehicle; SB= SB203580

Figure S2: Time in the three compartments of the CPP during pretest. Overall, there was no difference in the time spent between the vertical and the horizontal compartments of the CPP in any of the groups used in the study. Statistical test: one‐way ANOVA, effect: time spent in compartments, followed by Tukey's post hoc test. Pretest future Sal CTR, Vertical vs horizontal, p=0.8274; Pretest future cocaine, Vertical vs horizontal, p=0.9996; Pretest future SI, Vertical vs horizontal, p=0.9986; Pretest future Sal CTR – with surgery, Vertical vs horizontal, p=0.4983; Pretest future cocaine – with surgery, Vertical vs horizontal, p=0.9871; Pretest future SI‐ with surgery, Vertical vs horizontal, p=0.8359. Control=Sal CTR; SI= Social CPP.

Table S1: CRF, CRFR1 and CRFR2 expression in the hypothalamus and the amygdala 24 hours after the CPP test (n=4). Naïve rats are untreated rats. Control=Sal CTR; COC= cocaine CPP; SI= Social CPP

Table S2: List of Primers used in the q‐PCR experiment.

## References

[1] Fritz M , El Rawas R , Salti A , et al. Reversal of cocaine‐conditioned place preference and mesocorticolimbic Zif268 expression by social interaction in rats. Addict Biol. 2011;16(2):273‐284. 10.1111/j.1369-1600.2010.00285.x 21309948

[2] Bregolin T , Pinheiro BS , El Rawas R , Zernig G . Preventive strength of dyadic social interaction against reacquisition/reexpression of cocaine conditioned place preference. Front Behav Neurosci. 2017;11:225 10.3389/fnbeh.2017.00225 29167636 PMC5682322

[3] Ribeiro Do Couto B , Aguilar MA , Lluch J , Rodríguez‐Arias M , Miñarro J . Social experiences affect reinstatement of cocaine‐induced place preference in mice. Psychopharmacology (Berl). 2009;207(3):485‐498. 10.1007/s00213-009-1678-1 19798482

[4] Le Moal M . Drug abuse: vulnerability and transition to addiction. Pharmacopsychiatry. 2009;42(S 01):S42‐S55. 10.1055/s-0029-1216355 19434555

[5] Sinha R , Shaham Y , Heilig M . Translational and reverse translational research on the role of stress in drug craving and relapse. Psychopharmacology (Berl). 2011;218(1):69‐82. 10.1007/s00213-011-2263-y 21494792 PMC3192289

[6] Logrip ML , Koob GF , Zorrilla EP . Role of corticotropin‐releasing factor in drug addiction: potential for pharmacological intervention. CNS Drugs. 2011;25(4):271‐287. 10.2165/11587790-000000000-00000 21425881 PMC3273042

[7] Cador M , Dumas S , Cole BJ , et al. Behavioral sensitization induced by psychostimulants or stress: search for a molecular basis and evidence for a CRF‐dependent phenomenon. Ann N Y Acad Sci. 1992;654(1):416‐420. 10.1111/j.1749-6632.1992.tb25985.x 1352953

[8] Erb S , Funk D , Lê AD . Prior, repeated exposure to cocaine potentiates locomotor responsivity to central injections of corticotropin‐releasing factor (CRF) in rats. Psychopharmacology (Berl). 2003;170(4):383‐389. 10.1007/s00213-003-1556-1 12955298

[9] Kalivas PW , Duffy P , Latimer LG . Neurochemical and behavioral effects of corticotropin‐releasing factor in the ventral tegmental area of the rat. J Pharmacol Exp Ther. 1987;242(3):757‐763. http://www.ncbi.nlm.nih.gov/pubmed/3498816 3498816

[10] Lu L , Liu D , Ceng X . Corticotropin‐releasing factor receptor type 1 mediates stress‐induced relapse to cocaine‐conditioned place preference in rats. Eur J Pharmacol. 2001;415(2‐3):203‐208. 10.1016/s0014-2999(01)00840-8 11275000

[11] Shaham Y , Funk D , Erb S , Brown TJ , Walker CD , Stewart J . Corticotropin‐releasing factor, but not corticosterone, is involved in stress‐induced relapse to heroin‐seeking in rats. J Neurosci. 1997;17(7):2605‐2614. http://www.ncbi.nlm.nih.gov/pubmed/9065520 9065520 10.1523/JNEUROSCI.17-07-02605.1997 PMC6573519

[12] Erb S , Shaham Y , Stewart J . The role of corticotropin‐releasing factor and corticosterone in stress‐ and cocaine‐induced relapse to cocaine seeking in rats. J Neurosci. 1998;18(14):5529‐5536. http://www.ncbi.nlm.nih.gov/pubmed/9651233 9651233 10.1523/JNEUROSCI.18-14-05529.1998 PMC6793509

[13] Spealman RD , Lee B , Tiefenbacher S , Platt DM , Rowlett JK , Khroyan TV . Triggers of relapse: nonhuman primate models of reinstated cocaine seeking. Nebr Symp Motiv. 2004;50:57‐84. http://www.ncbi.nlm.nih.gov/pubmed/15160638 15160638

[14] Rodaros D , Caruana DA , Amir S , Stewart J . Corticotropin‐releasing factor projections from limbic forebrain and paraventricular nucleus of the hypothalamus to the region of the ventral tegmental area. Neuroscience. 2007;150(1):8‐13. 10.1016/j.neuroscience.2007.09.043 17961928

[15] Korosi A , Baram TZ . The central corticotropin releasing factor system during development and adulthood. Eur J Pharmacol. 2008;583(2‐3):204‐214. 10.1016/j.ejphar.2007.11.066 18275957 PMC2329668

[16] Zheng G , Chen Y , Zhang X , et al. Acute cold exposure and rewarming enhanced spatial memory and activated the MAPK cascades in the rat brain. Brain Res. 2008;1239:171‐180. 10.1016/j.brainres.2008.08.057 18789908

[17] Bruchas MR , Land BB , Aita M , et al. Stress‐induced p38 mitogen‐activated protein kinase activation mediates‐opioid‐dependent dysphoria. J Neurosci. 2007;27(43):11614‐11623. 10.1523/JNEUROSCI.3769-07.2007 17959804 PMC2481272

[18] Sanchez MM , Alagbe O , Felger JC , et al. Activated p38 MAPK is associated with decreased CSF 5‐HIAA and increased maternal rejection during infancy in rhesus monkeys. Mol Psychiatry. 2007;12(10):895‐897. 10.1038/sj.mp.4002025 17895923

[19] Zhang X‐Q , Cui Y , Cui Y , et al. Activation of p38 signaling in the microglia in the nucleus accumbens contributes to the acquisition and maintenance of morphine‐induced conditioned place preference. Brain Behav Immun. 2012;26(2):318‐325. 10.1016/j.bbi.2011.09.017 22004988

[20] Gerdjikov TV , Ross GM , Beninger RJ . Place preference induced by nucleus accumbens amphetamine is impaired by antagonists of ERK or p38 MAP kinases in rats. Behav Neurosci. 2004;118(4):740‐750. 10.1037/0735-7044.118.4.740 15301601

[21] Salti A , Kummer KK , Sadangi C , Dechant G , Saria A , El Rawas R . Social interaction reward decreases p38 activation in the nucleus accumbens shell of rats. Neuropharmacology. 2015;99:510‐516. 10.1016/j.neuropharm.2015.08.029 26300300 PMC5056637

[22] Kalueff AV , Tuohimaa P . The grooming analysis algorithm discriminates between different levels of anxiety in rats: potential utility for neurobehavioural stress research. J Neurosci Methods. 2005;143(2):169‐177. 10.1016/j.jneumeth.2004.10.001 15814150

[23] Kalueff AV , Aldridge JW , LaPorte JL , Murphy DL , Tuohimaa P . Analyzing grooming microstructure in neurobehavioral experiments. Nat Protoc. 2007;2(10):2538‐2544. 10.1038/nprot.2007.367 17947996

[24] Shaham Y , Funk D , Erb S , Brown TJ , Walker CD , Stewart J . Corticotropin‐releasing factor, but not corticosterone, is involved in stress‐induced relapse to heroin‐seeking in rats. J Neurosci. 1997;17(7):2605‐2614. http://www.ncbi.nlm.nih.gov/pubmed/9065520 9065520 10.1523/JNEUROSCI.17-07-02605.1997 PMC6573519

[25] Blanchard DC , Sakai RR , McEwen B , Weiss SM , Blanchard RJ . Subordination stress: behavioral, brain, and neuroendocrine correlates. Behav Brain Res. 1993;58(1‐2):113‐121. http://www.ncbi.nlm.nih.gov/pubmed/8136039 8136039 10.1016/0166-4328(93)90096-9

[26] Veenema AH , Neumann ID . Maternal separation enhances offensive play‐fighting, basal corticosterone and hypothalamic vasopressin mRNA expression in juvenile male rats. Psychoneuroendocrinology. 2009;34(3):463‐467. 10.1016/j.psyneuen.2008.10.017 19056182

[27] Nyuyki KD , Beiderbeck DI , Lukas M , Neumann ID , Reber SO . Chronic subordinate colony housing (CSC) as a model of chronic psychosocial stress in male rats. PLoS ONE. 2012;7(12):e52371 10.1371/journal.pone.0052371 23300653 PMC3530595

[28] Niebylski A , Boccolini A , Bensi N , et al. Neuroendocrine changes and natriuresis in response to social stress in rats. Stress Health. 2012;28(3):179‐185. 10.1002/smi.1411 22282077

[29] Mann EA , Alam Z , Hufgard JR , et al. Chronic social defeat, but not restraint stress, alters bladder function in mice. Physiol Behav. 2015;150:83‐92. 10.1016/j.physbeh.2015.02.021 25689100 PMC4537697

[30] Funk D , Li Z , Lê AD . Effects of environmental and pharmacological stressors on c‐fos and corticotropin‐releasing factor mRNA in rat brain: relationship to the reinstatement of alcohol seeking. Neuroscience. 2006;138(1):235‐243. 10.1016/j.neuroscience.2005.10.062 16359808

[31] Fekete EM , Zhao Y , Li C , Sabino V , Vale WW , Zorrilla EP . Social defeat stress activates medial amygdala cells that express type 2 corticotropin‐releasing factor receptor mRNA. Neuroscience. 2009;162(1):5‐13. 10.1016/j.neuroscience.2009.03.078 19358876 PMC2754763

[32] McReynolds JR , Vranjkovic O , Thao M , et al. Beta‐2 adrenergic receptors mediate stress‐evoked reinstatement of cocaine‐induced conditioned place preference and increases in CRF mRNA in the bed nucleus of the stria terminalis in mice. Psychopharmacology (Berl). 2014;231(20):3953‐3963. 10.1007/s00213-014-3535-0 24696080 PMC8647032

[33] Cador M , Ahmed SH , Koob GF , Le Moal M , Stinus L . Corticotropin‐releasing factor induces a place aversion independent of its neuroendocrine role. Brain Res. 1992;597(2):304‐309. http://www.ncbi.nlm.nih.gov/pubmed/1473001 1473001 10.1016/0006-8993(92)91487-y

[34] Nader J , Claudia C , El Rawas R , et al. Loss of environmental enrichment increases vulnerability to cocaine addiction. Neuropsychopharmacology. 2012;37(7):1579‐1587. 10.1038/npp.2012.2 22334125 PMC3358749

[35] Erb S , Stewart J . A role for the bed nucleus of the stria terminalis, but not the amygdala, in the effects of corticotropin‐releasing factor on stress‐induced reinstatement of cocaine seeking. J Neurosci. 1999;19(20):1‐6, RC35. http://www.ncbi.nlm.nih.gov/pubmed/10516337 10516337 10.1523/JNEUROSCI.19-20-j0006.1999 PMC6782764

[36] Lu L , Liu Z , Huang M , Zhang Z . Dopamine‐dependent responses to cocaine depend on corticotropin‐releasing factor receptor subtypes. J Neurochem. 2003;84(6):1378‐1386. http://www.ncbi.nlm.nih.gov/pubmed/12614338 12614338 10.1046/j.1471-4159.2003.01635.x

[37] Corominas M , Roncero C , Casas M . Corticotropin releasing factor and neuroplasticity in cocaine addiction. Life Sci. 2010;86(1‐2):1‐9. 10.1016/j.lfs.2009.11.005 19914260

[38] Kreibich AS , Briand L , Cleck JN , Ecke L , Rice KC , Blendy JA . Stress‐induced potentiation of cocaine reward: a role for CRF R1 and CREB. Neuropsychopharmacology. 2009;34(12):2609‐2617. 10.1038/npp.2009.91 19675537 PMC4034179

[39] Kasahara M , Groenink L , Bijlsma EY , Olivier B , Sarnyai Z . Lifelong, central corticotropin‐releasing factor (CRF) overexpression is associated with individual differences in cocaine‐induced conditioned place preference. Eur J Pharmacol. 2015;753:151‐157. 10.1016/j.ejphar.2014.07.050 25094033

[40] Campbell BM , Morrison JL , Walker EL , Merchant KM . Differential regulation of behavioral, genomic, and neuroendocrine responses by CRF infusions in rats. Pharmacol Biochem Behav. 2004;77(3):447‐455. 10.1016/j.pbb.2003.12.010 15006454

[41] Hostetler CM , Ryabinin AE . The CRF system and social behavior: a review. Front Neurosci. 2013;7:92 10.3389/fnins.2013.00092 23754975 PMC3668170

[42] Millan MJ , Brocco M , Gobert A , Dorey G , Casara P , Dekeyne A . Anxiolytic properties of the selective, non‐peptidergic CRF(1) antagonists, CP154,526 and DMP695: a comparison to other classes of anxiolytic agent. Neuropsychopharmacology. 2001;25(4):585‐600. 10.1016/S0893-133X(01)00244-5 11557172

[43] Maciag CM , Dent G , Gilligan P , et al. Effects of a non‐peptide CRF antagonist (DMP696) on the behavioral and endocrine sequelae of maternal separation. Neuropsychopharmacology. 2002;26(5):574‐582. 10.1016/S0893-133X(01)00398-0 11927182

[44] Lukkes J , Vuong S , Scholl J , Oliver H , Forster G . Corticotropin‐releasing factor receptor antagonism within the dorsal raphe nucleus reduces social anxiety‐like behavior after early‐life social isolation. J Neurosci. 2009;29(32):9955‐9960. 10.1523/JNEUROSCI.0854-09.2009 19675229 PMC2772211

[45] Venniro M , Zhang M , Caprioli D , et al. Volitional social interaction prevents drug addiction in rat models. Nat Neurosci. 2018;21(11):1520‐1529. 10.1038/s41593-018-0246-6 30323276 PMC7386559

[46] Sampedro‐Piquero P , Ávila‐Gámiz F , Moreno Fernández RD , Castilla‐Ortega E , Santín LJ . The presence of a social stimulus reduces cocaine‐seeking in a place preference conditioning paradigm. J Psychopharmacol. September. 2019;33(12):1501‐1511. 10.1177/0269881119874414 31542987

[47] Mannangatti P , NarasimhaNaidu K , Damaj MI , Ramamoorthy S , Jayanthi LD . A role for p38 mitogen‐activated protein kinase‐mediated threonine 30‐dependent norepinephrine transporter regulation in cocaine sensitization and conditioned place preference. J Biol Chem. 2015;290(17):10814‐10827. 10.1074/jbc.M114.612192 25724654 PMC4409246

[48] Sinha R , Jastreboff AM . Stress as a common risk factor for obesity and addiction. Biol Psychiatry. 2013;73(9):827‐835. 10.1016/j.biopsych.2013.01.032 23541000 PMC3658316

[49] Southwick SM , Sippel L , Krystal J , Charney D , Mayes L , Pietrzak R . Why are some individuals more resilient than others: the role of social support. World Psychiatry. 2016;15(1):77‐79. 10.1002/wps.20282 26833614 PMC4780285

[50] Rudzinski K , McDonough P , Gartner R , Strike C . Is there room for resilience? A scoping review and critique of substance use literature and its utilization of the concept of resilience. Subst Abuse Treat Prev Policy. 2017;12(1):41 10.1186/s13011-017-0125-2 28915841 PMC5603070

