# Supplementary figures and images for: Social interaction reward in rats has anti‐stress effects
Source: Addict Biol. 2020 Jan 26;26(1):e12878. doi: 10.1111/adb.12878 (PMC7757251; doi:10.1111/adb.12878)

## Concurrent CPP

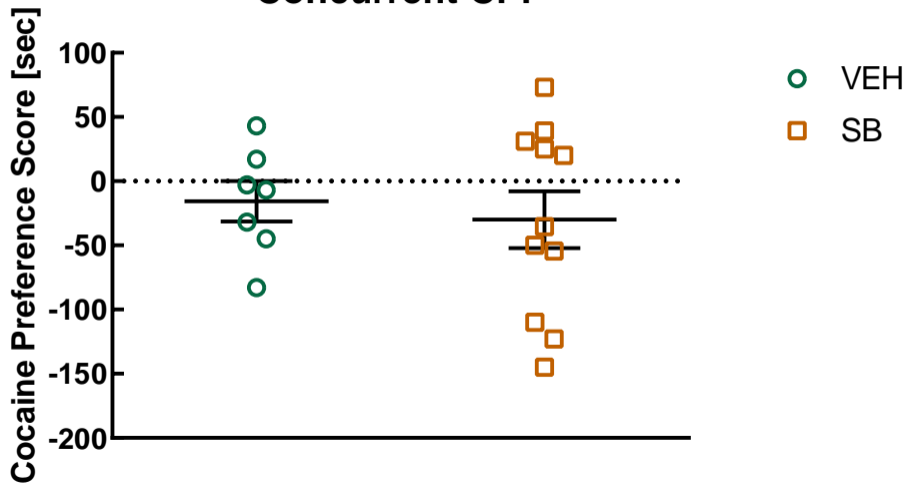

Supplement: Supplementary file 1 — Figure S1: Effects of icv injections of SB203580 on concurrent CPP (n=7‐11). Rats received icv injection of either VEH or SB before each cocaine and social interaction conditioning. Preference score is the time that the rat spent in the cocaine‐associated compartment during the test – pretest. VEH (n=7); SB (n=11). Statistical test two‐tailed unpaired t‐test. VEH= Vehicle; SB= SB203580 [file ADB-26-e12878-s001.pdf]
