# Supplementary material for: Social interaction reward in rats has anti‐stress effects
Source: Addict Biol. 2020 Jan 26;26(1):e12878. doi: 10.1111/adb.12878 (PMC7757251; doi:10.1111/adb.12878)
Supplement: Supplementary file 2 — Figure S2: Time in the three compartments of the CPP during pretest. Overall, there was no difference in the time spent between the vertical and the horizontal compartments of the CPP in any of the groups used in the study. Statistical test: one‐way ANOVA, effect: time spent in compartments, followed by Tukey's post hoc test. Pretest future Sal CTR, Vertical vs horizontal, p=0.8274; Pretest future cocaine, Vertical vs horizontal, p=0.9996; Pretest future SI, Vertical vs horizontal, p=0.9986; Pretest future Sal CTR – with surgery, Vertical vs horizontal, p=0.4983; Pretest future cocaine – with surgery, Vertical vs horizontal, p=0.9871; Pretest future SI‐ with surgery, Vertical vs horizontal, p=0.8359. Control=Sal CTR; SI= Social CPP. [file ADB-26-e12878-s002.pdf]

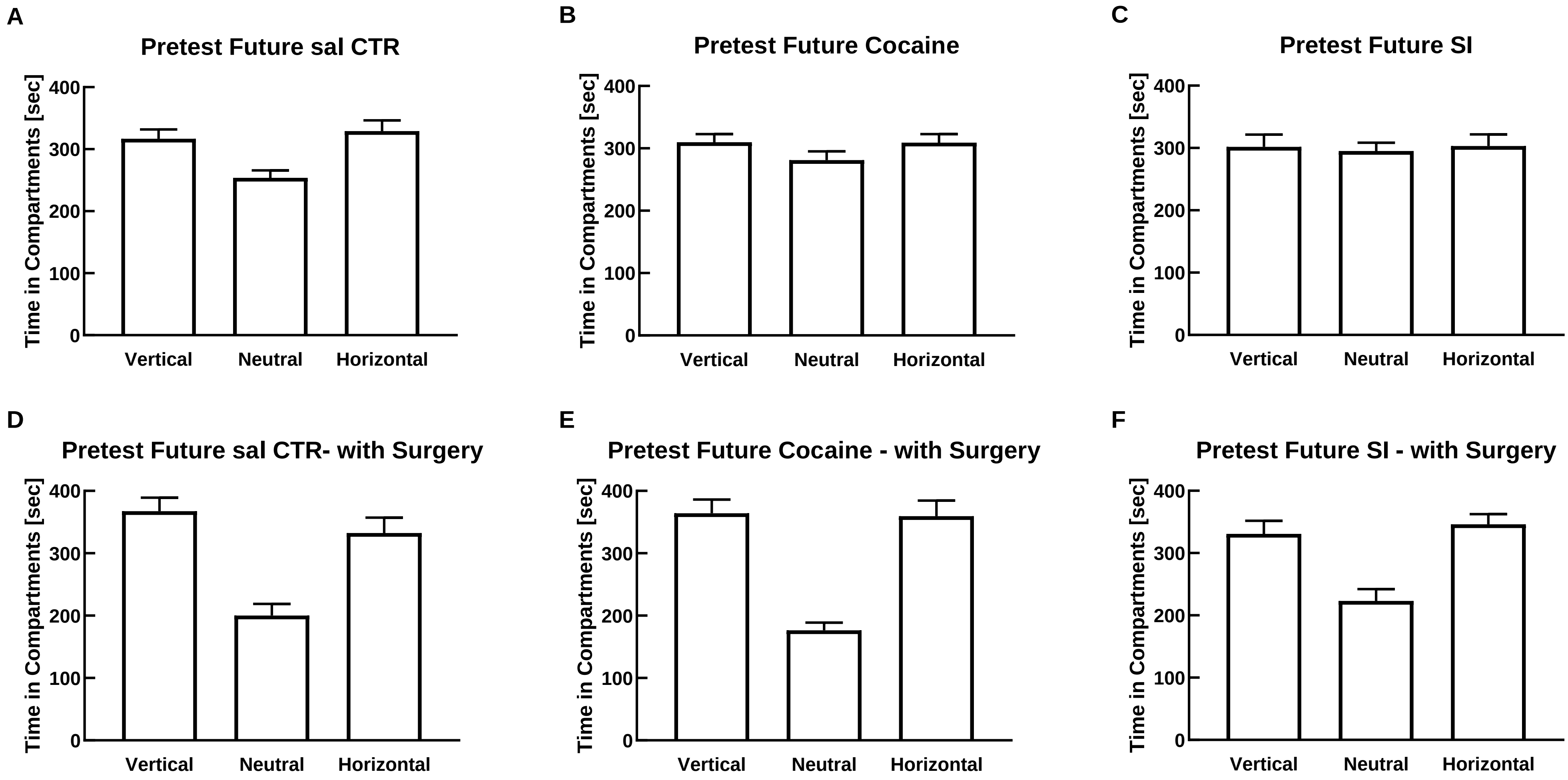

A
Pretest Future sal CTR
B
Pretest Future Cocaine
C
Pretest Future SI
D
Pretest Future sal CTR- with Surgery
E
Pretest Future Cocaine - with Surgery
F
Pretest Future SI - with Surgery
Time in Compartments [sec]
0
100
200
300
400
Vertical
Neutral
Horizontal
