# Supplementary material for: Social interaction reward in rats has anti‐stress effects
Source: Addict Biol. 2020 Jan 26;26(1):e12878. doi: 10.1111/adb.12878 (PMC7757251; doi:10.1111/adb.12878)
Supplement: Supplementary file 3 — Table S1: CRF, CRFR1 and CRFR2 expression in the hypothalamus and the amygdala 24 hours after the CPP test (n=4). Naïve rats are untreated rats. Control=Sal CTR; COC= cocaine CPP; SI= Social CPP [file ADB-26-e12878-s003.pdf]

Treatment	Naive	Sal CTR	COC	SI
CRF	1.000 ± 0.041	1.000 ± 0.041	0.850 ± 0.233	0.825 ± 0.359
CRFR1	1.075 ± 0.085	1.075 ± 0.048	1.150 ± 0.087	0.850 ± 0.029
CRFR2	1.075 ± 0.111	0.975 ± 0.085	0.875 ± 0.165	1.075 ± 0.269

Region: Hypothalamus. Data are shown as mean relative mRNA Level ± SEM

Treatment	Naive	Sal CTR	COC	SI
CRF	0.875 ± 0.111	0.975 ± 0.085	0.800 ± 0.108	0.925 ± 0.160
CRFR1	0.975 ± 0.111	1.000 ± 0.091	1.050 ± 0.272	1.000 ± 0.227
CRFR2	1.275 ± 0.232	1.050 ± 0.171	1.275 ± 0.427	1.125 ± 0.250

Region: Amygdala. Data are shown as mean relative mRNA Level ± SEM
