# Supplementary material for: Social interaction reward in rats has anti‐stress effects
Source: Addict Biol. 2020 Jan 26;26(1):e12878. doi: 10.1111/adb.12878 (PMC7757251; doi:10.1111/adb.12878)
Supplement: Supplementary file 4 — Table S2: List of Primers used in the q‐PCR experiment. [file ADB-26-e12878-s004.pdf]

Primers	Forward (5'-3')	Reverse (5'-3')
rCRF	TCGGCTGTCCCCCAACTC	CTGCAGCAACACGCGGAAAA
rCRFR1	GTGAGGTCCGCTCCGCTA	ACTGCTGTGGACTGCTTGATG
rCRFR2	GCTTCTTCAATGGAGAGGTGC	AGCTGTCTGCTTGATGCTGTG
rp38	GAAGAGCCTGACCTACGATG	GCCTTCCCCTCACAGTGAA
